# Receipt of COVID-19 emergency funds and engagement in sex work during COVID-19 among people who use drugs: evidence from Vancouver, Canada

**DOI:** 10.1186/s12954-024-00997-w

**Published:** 2024-04-27

**Authors:** Erica McAdam, Kanna Hayashi, Zishan Cui, Haleigh Anderson, Scarlett Nelson, M.-J. Milloy, Kora DeBeck

**Affiliations:** 1 British Columbia Centre on Substance Use, Howe St, 400-1045, V6Z 2A9 Vancouver, BC Canada; 2 Faculty of Health Sciences, Simon Fraser University, 8888 University Drive, V5A 1S6 Burnaby, BC Canada; 3 Department of Medicine, University of British Columbia, St. Paul’s Hospital, 1081 Burrard Street, 608–, V6Z 1Y6 Vancouver, BC Canada; 4 Peer Research Associates, At-Risk Youth Study, 1265 Granville St, V6Z 1M5 Vancouver, BC Canada; 5 School of Public Policy, Simon Fraser University, 515 West Hastings St, V6B 5K3 Vancouver, BC Canada; 6 CIHR Applied Public Health Chair, Simon Fraser University, 515 West Hastings St, V6B 5K3 Vancouver, BC Canada

**Keywords:** Sex work, Sex trade, COVID-19, Gender, Income generation, Substance use

## Abstract

**Background:**

During the early period of the COVID-19 pandemic, public health orders disrupted income generation in numerous sectors and many governments provided emergency financial support. Access to government support and changes in engagement in sex work during the early period of the pandemic among people who use drugs (PWUD) are not well described. In the present study, we investigate the prevalence and correlates of engaging in sex work during the COVID-19 pandemic, among PWUD in Vancouver, Canada.

**Methods:**

Data derived from three harmonized cohorts of PWUD. Using multivariable logistic regression, we characterized factors associated with engaging in sex work in the last month between July 17 and November 30, 2020. Reports of changes in frequency of engagement in sex work since the pandemic were also collected.

**Results:**

Of the 864 individuals included in this analysis, 55 (6.4%) reported sex work engagement in the last month. Among these participants, 40.7% reported receiving COVID-19 income support in the past month vs. 52.7% of the rest of the sample, though receipt of income support in the past six months was similar between the two groups (72.2% vs. 75.7%, *p* = 0.624). In multivariable analysis, receipt of financial support in the last month was negatively associated with engagement in sex work in the last month (adjusted odds ratio [AOR] = 0.44 [95% confidence interval [CI]: 0.24–0.81]). Among 69 participants who responded to a question regarding changes in engagement in sex work, 38 (55.1%) reported a decrease, 11 (15.9%) reported an increase, 19 (27.5%) reported no change, and 1 (1.4%) reported cessation.

**Conclusions:**

Findings document that engagement in sex work appears to have declined early in the pandemic. Participants who received income support in the past month were less likely to report recent engagement in sex work. Findings suggest that recent receipt of income support may have contributed to reductions in engagement in sex work. Additional investigation is warranted.

## Introduction

In the immediate wake of the emergence of the novel SARS-CoV-2 coronavirus, many governments around the world enacted wide-ranging measures to prevent viral transmission, including closures of non-essential businesses and physical distancing requirements. Economic policies have been enacted during the COVID-19 pandemic to provide financial support for citizens and businesses financially affected by pandemic restrictions. In our Canadian context, the federal government provided various financial support measures, including the *Canadian Emergency Response Benefit* (CERB), which provided emergency financial support to workers who were affected by the pandemic and specific supports for businesses, such as rent subsidies [1]. Additionally, provincial governments implemented additional financial supports for both citizens and businesses.

As a result of COVID-19 pandemic-induced decline in demand and restrictions to prevent the spread of the virus, sex work has been documented to have largely declined in both the formal and non-formal economies during the early period of the pandemic [2–8]. In the same study context, research conducted by Pearson et al. from April 2020 to April 2021 focusing on people engaged in sex work in Vancouver, Canada, found that 23.6% reported they stopped engaging in sex work since the start of the pandemic and 71.6% reported reduced income from in-person sex work [9]. Importantly, people engaged in sex work who engage in survival sex work or who were financially precarious had a higher risk of becoming financially compromised during the pandemic. Research conducted in Vancouver in 2014 estimated that among 2,600 people engaged in sex work in the city, approximately 20% engaged in survival sex work, defined as using income to pay for necessities like food, shelter, and drugs [10]. Moreover, preliminary research focused on people engaged in sex work located in the Downtown Eastside (DTES) of Vancouver—an urban neighbourhood with high rates of substance use, criminalization and marginalization—reported that a substantial portion experienced difficulty paying for food, medications, rent, and other essentials during the pandemic and faced barriers to accessing governmental income supports [7, 9, 11]. It is less clear, however, what the impact of COVID-19-related changes on sex work had among people who use drugs (PWUD). To address this gap, we sought to characterize the prevalence and correlates of engaging in sex work and assess reports of changes in the frequency of sex work engagement during the early period of the COVID-19 pandemic (July to November 2020) among cohorts of PWUD in Vancouver, Canada. Understanding the experiences of people engaged in sex work who use drugs during the pandemic can inform future pandemic responses to support this uniquely structurally marginalized group.

## Methods

Data for this study were derived from three ongoing prospective cohorts of PWUD in Vancouver, Canada: the At-Risk Youth Study (ARYS), the Vancouver Injection Drug Users Study (VIDUS), and the AIDS Care Cohort to Evaluate exposure to Survival Services (ACCESS). Details of these studies and their harmonized procedures have been described in detail previously and published elsewhere [12–14]. In brief, to be eligible for enrolment, participants must have used drugs (other than or in addition to cannabis) within the previous 30 days, reside in the Greater Vancouver area, and provide written informed consent. The ARYS cohort includes participants between the ages of 14–26 at enrolment and are “street-involved”, defined as being without stable housing or utilizing services for youth experiencing homelessness within the last month. The VIDUS cohort includes participants who are 18 years or older, are HIV-negative and have injected drugs within the last month. The ACCESS cohort includes participants 18 years or older living with HIV.

Participants from all three cohorts complete an interviewer-administered questionnaire at baseline and every six months thereafter. The questionnaire collects data on demographics, substance use patterns and associated risks, income generation activities, and health and social service engagement, among others. All participants receive a $40 CAD honorarium at each study visit.

Due to the COVID-19 pandemic, all in-person data collection activities were halted in March 2020. New COVID-19 safe study protocols were developed, and the study instrument was revised to include items specific to the COVID-19 pandemic. On July 17, 2020, data collection for those already enrolled in the cohorts was resumed. Study interviews were no longer hosted in person but were conducted remotely via telephone or videoconferencing. Participants were initially contacted via telephone and social media to inform them they were due for a remote study follow-up visit. Those interested in participating were provided with the option to complete the interview via telephone or videoconferencing. Participants who did not have access to a telephone were provided with the option to utilize a study-owned pre-paid cellphone for the purposes of conducting the interview. If a participant did not have access to a private space to complete the interview, a space was provided for them to use. Participants with access to online banking or a bank account received their honorarium via e-transfer. For participants who did not have access to a bank account, arrangements were made to pick up the cash honorarium in person. The University of British Columbia/Providence Health Care Research Ethics Board has approved all cohorts.

Participants who completed an interview between July 17 to November 30, 2020, were included in the present study. These dates correspond to one modified follow-up round in the cohorts (typical follow-up cycles are Dec 1 – May 31 and June 1 – November 30). The primary outcome for this study was self-reported engagement in sex work in the previous month (yes vs. no). Engagement in sex work was defined as exchanging sex for money, goods, drugs, shelter, or anything else during the previous month. Except for time-fixed variables or, unless otherwise specified, all variables referred to the month prior to the interview. Variables hypothesized to be associated with our outcome of interest included: self-identified gender (woman or self-described gender vs. man—woman or self-described gender category included participants who identify as transgender, Two-Spirit, and those who preferred to self-describe in an ‘other’ category); age (per year older); race/ancestry (Black, Indigenous, or other Persons of Colour (BIPOC) vs. white); educational attainment (< high school diploma vs. ≥ high school diploma); experiencing homelessness in the past six months, defined as having no fixed address, sleeping on the street, or staying in a shelter or hostel (yes vs. no); regular employment, defined as having a regular job, temporary work or self-employed (yes vs. no); receipt of COVID-19 emergency aid funds (yes vs. no); at least weekly drug injection (yes vs. no); at least weekly cocaine use (yes vs. no); at least weekly crack cocaine use (yes vs. no); at least weekly heroin/fentanyl/down use (yes vs. no); at least weekly crystal methamphetamine use (yes vs. no); at least weekly non-medical prescription opioid (PO) use, defined as taking POs that were not prescribed or taking POs only for the experience or feeling they caused (yes vs. no) [15, 16]; non-fatal overdose, defined as an acute reaction or overdose following drug use (yes vs. no); and involvement in drug dealing, defined as receiving money in exchange for drugs (yes vs. no).

As a first step in the analysis, the bivariable associations between our primary outcome of interest and independent variables of interest were estimated using logistic regression. Variables of interest that were associated with engaging in sex work at the *p* < 0.10 threshold in the bivariable analysis were used to construct a multivariable model based on the Akaike information criterion with the best subset selection procedure [17].

Participants were also asked if their frequency of engagement in sex changed since the COVID-19 pandemic using the question, “has your level of engagement in sex work changed since COVID-19 began?” (decreased vs. increased vs. no change vs. stopped engagement). The time period for the start of the COVID-19 emergency was not specified in the question; however, we consider the start of the pandemic to be March 2020 within the Canadian context. Additionally, data was collected on receipt of COVID-19 emergency funds in the past six months. COVID-19 funds were limited in the time that they were available. For example, CERB was available from March 15 to December 2, 2020 [18], and the Province of BC’s $300 per month top-up on income and disability support payments was distributed to eligible people between April and December 2020 [19]. Data was also collected on receipt of COVID-19 emergency funds in the past six months, however, this was used only for descriptive and comparative purposes and not utilized in statistical analyses to ensure that the recall period for variables of interest was consistent.

All statistical analyses were performed using SAS software version 9.4 [20]. All *p*-values are two-sided and considered statistically significant at *p* < 0.05.

## Results

Overall, 884 participants were seen for a study visit between July 17 and November 30, 2020. There were 20 missing values for our outcome of interest (engagement in sex work in the past month). These individuals were excluded from the analysis. Characteristics of study participants stratified by engagement in sex work in the last month are shown in Table 1. Among the remaining 864 participants included, 317 (36.7%) identified as woman and 23 (2.7%) identified as transgender, Two-Spirit, or have self-described their gender, and 360 (43.7%) identified as Black, Indigenous or a Person of Colour. The median participant age was 46 years (first to third quartile: 33–56).

**Table 1:** Characteristics of study participants stratified by engagement in sex work in the last month (*n* = 864)

Characteristic	Engagement in Sex Work*	
	Yes (%) (*n* = 55)	No (%) (*n* = 809)
Age^†^ (median, Q1-Q3)	40 (33–49)	47 (33–56)
Woman, transgender, Two-Spirit, or other non-binary gender	40 (81.63)	300 (38.66)
BIPOC	31 (63.27)	329 (42.51)
< High school	33 (67.35)	384 (50.33)
Homelessness in last 6 months	9 (16.36)	108 (13.47)
Employment*	15 (27.27)	205 (25.53)
COVID-19 Funds*	22 (40.74)	404 (52.67)
≥ Weekly injection drug use*	35 (63.64)	281 (34.73)
≥ Weekly crack use*	18 (32.73)	142 (17.68)
≥ Weekly cocaine use*	3 (5.45)	46 (5.73)
≥ Weekly crystal meth use*	31 (56.36)	197 (24.53)
≥ Weekly heroin/fentanyl/down use*	40 (72.73)	288 (35.82)
≥ Weekly non-medical PO use*	2 (3.70)	17 (2.18)
Drug dealing*	23 (41.82)	150 (18.63)
Non-fatal overdose*	6 (10.91)	38 (4.76)

† Per one-year increase.

Q1-Q3 = first to third quartile.

* Activities reported in the last month.

Overall, 55 (6.4%) individuals reported engagement in sex work in the month prior to their interview. A total of 426 (51.9%) participants indicated that they received some form of COVID-19 emergency funds in the past month, while 644 (75.5%) reported receipt of emergency funds in the previous six months. Among participants engaged in sex work, 40.7% reported receiving COVID-19 income support in the past month vs. 52.7% of the rest of the sample. Receipt of income support in the past six months was similar between those engaged in sex work and the rest of the sample (72.2% vs. 75.7%, *p* = 0.624).

The results of the bivariable and multivariable analysis are shown in Table 2. In bivariate analysis, those who self-identified as women, transgender, or non-binary genders (odds ratio [OR] = 7.14, [95% CI: 3.33–14.29]); were Black, Indigenous, or a Person of Colour (OR = 2.33, 95% CI: 1.28–4.24); had less than high school education (OR = 2.04, [95% CI: 1.10–3.70]); engaged in at least weekly injection drug use (OR = 3.29, [95% CI: 1.86–5.80]); at least weekly crack use (OR = 2.26, [95% CI: 1.25–4.09]); at least weekly crystal methamphetamine use (OR = 3.97, [95% CI: 2.28–6.93]); at least weekly heroin/fentanyl/down use (OR = 4.78, [95% CI: 2.59–8.80]); and involvement in drug dealing (OR = 3.14, [95% CI: 1.78–5.52]) had an increased odds of reporting engagement in sex work during the COVID-19 pandemic.

**Table 2:** Bivariate and multivariable logistic regression analysis of factors associated with engagement in sex work in the last month (*n* = 864)

	Unadjusted		Adjusted	
Characteristic	Odds Ratio (95% CI)	p - value	Odds Ratio (95% CI)	p - value
Age^†^ (median, Q1-Q3)	0.98 (0.96-1.00)	0.026		
Self-identified gender				
(woman^‡^ vs. man)	7.14 (3.33–14.29)	< 0.0001	7.14 (3.33–16.67)	< 0.0001
Race/ancestry				
(BIPOC vs. white)	2.33 (1.28–4.24)	0.006		
Education				
(< high school vs. ≥ high school)	2.04 (1.10–3.70)	0.023		
Homelessness in last 6 months				
(yes vs. no)	1.26 (0.60–2.64)	0.546		
Employment*				
(yes vs. no)	1.09 (0.59–2.02)	0.775		
COVID-19 funds*				
(yes vs. no)	0.62 (0.35–1.08)	0.092	0.44 (0.24–0.81)	0.008
≥ Weekly injection drug use*				
(yes vs. no)	3.29 (1.86–5.80)	< 0.0001		
≥ Weekly crack use*				
(yes vs. no)	2.26 (1.25–4.09)	0.007	2.43 (1.25–4.73)	0.009
≥ Weekly cocaine use*				
(yes vs. no)	0.95 (0.29–3.16)			
≥ Weekly crystal meth use*				
(yes vs. no)	3.97 (2.28–6.93)	< 0.0001	3.44 (1.81–6.54)	0.000
≥ Weekly heroin/fentanyl use*				
(yes vs. no)	4.78 (2.59–8.80)	< 0.0001	3.15 (1.61–6.15)	0.001
≥ Weekly non-medical PO use*				
(yes vs. no)	1.73 (0.39–7.67)	0.473		
Drug dealing*				
(yes vs. no)	3.14 (1.78–5.52)	< 0.0001		
Non-fatal overdose*				
(yes vs. no)	2.45 (0.99–6.08)	0.053		

† Per one-year increase

Q1-Q3 = first to third quartile

‡ Includes participants who identify as transgender, Two-Spirit and other non-binary genders

* Activities reported in the last month

In the multivariable analysis (AIC of full model: 342.822; AIC of final reduced model: 332.509), engagement in sex work during the COVID-19 pandemic was independently and negatively associated with reduced odds of reporting receipt of COVID-19 emergency funds in the past month (adjusted odds ratio [AOR] = 0.44 [95% CI: 0.24–0.81]). Factors that were independently and positively associated with an increased odds of reporting engagement in sex work during the pandemic included, being a self-identified women, transgender, Two-Spirit, or other non-binary gender (AOR = 7.14 [95% CI: 3.33–16.67]), engagement in at least weekly crack cocaine use (AOR = 2.43 [95% CI: 1.25–4.73]), at least weekly opioid use (AOR = 3.15 [95% CI: 1.16–6.15]), and at least weekly crystal methamphetamine use (AOR = 3.44 [95% CI: 1.81–6.54]). A total of 69 participants responded to the question regarding the change in frequency in engagement in sex work since the pandemic: 38 (55.1%) participants reported that their level of engagement in sex work had decreased since the COVID-19 pandemic, while 11 (15.9%) reported it had increased. 19 (27.5%) indicated that there had been no change to their level of engagement, and 1 (1.4%) indicated that they ceased engagement in sex work.

## Discussion

In this study of PWUD, we observe that sex work engagement appeared to have declined during the early period of the COVID-19 pandemic and was negatively associated with receipt of COVID-19 emergency funds in the last month. Since the COVID-19 pandemic began, over half of the participants who engaged in sex work indicated that their level of engagement in had decreased, while 16% reported it had increased. Although participants who reported engaging in sex work were less likely to have received COVID-19 emergency funds in the last month, there were no significant differences in receipt of COVID-19 emergency funds in the last six months between those engaged in sex work vs. not (72.2% vs. 75.7%).

Although to our knowledge, this is the first study to investigate the prevalence of and factors associated with engagement in sex work during the COVID-19 pandemic among an exclusive sample of PWUD, studies undertaken prior to the COVID-19 pandemic among samples of PWUDs in the current study cohorts found the prevalence of sex work engagement in the last six months to range from 11 to 18% [21–23]. The suspected decline in engagement in sex work observed during the early period of the COVID-19 pandemic aligns with existing research among people engaged in sex work more generally [3, 4, 11, 24]. Also consistent with other studies, we observed that individuals who engage in high-intensity substance use were significantly more likely to be involved in sex work [21–23, 25–27]. Money to pay for drugs has been established to be an important factor in a PWUDs’ decision, particularly those with higher intensity substance use, to engage in sex work [28, 29]. Additionally, our research found that participants who identified as women, transgender, Two-Spirit, or other non-binary genders were significantly more likely to engage in sex work than men, which is also consistent with previous studies [25, 30].

One interpretation of the finding that PWUD engaged in sex work had a reduced odds of having received governmental income support, is that the receipt of COVID-19 emergency funds may have allowed some PWUD to potentially forgo engagement in sex work during the pandemic. Economic marginalization is endemic among PWUD which often requires individuals to resort to risky income generation, including sex work, for survival. Those engaged in sex work face harms which have been well characterized and stem from criminalization and stigma [31–33]. During the pandemic, people engaged in sex work faced heightened risks, including the risk of becoming infected with COVID-19; additionally, many individuals working in the sex trade experience multiple overlapping health inequalities, including barriers to accessing proper health care, which may exacerbate harms related to contracting COVID-19 [34–39]. Previous studies indicate that PWUD who are involved in sex work report a high degree of willingness to forgo sex work if they did not need money to purchase drugs [22] or if they had alternative low-threshold employment options [23]. Although this interpretation is consistent with previous studies highlighting that poverty and economic security impact PWUD’s decisions to engage in sex work [22, 23, 29, 40–42], the extent to which receipt of COVID-19 emergency funds may reduce risky income generation warrants additional investigation.

Another possible explanation of the findings from this study is that people engaged in sex work who use drugs may have experienced barriers to accessing government support, which would explain the negative association between sex work engagement and receipt of emergency funds. Previous research in Vancouver among people engaged in sex work found that less than half (48.6%) reported accessing income supports during the pandemic, which was significantly lower in comparison to other service workers (57–67%) [9]. Although the current study found a discrepancy between receipt of income support in the previous month for those engaged in sex work, only 28% of participants engaged in sex work reported not receiving COVID-19 funding in the past six months which was comparable to study participants who did not engage in sex work (24.3%) and notably lower than previous reports in the current study setting [9]. Within our study, people engaged in sex work did not seem to face more barriers to accessing income supports than people who use drugs more generally. This suggests that current receipt of emergency funds may contribute to a reduction in sex work engagement.

Consistent with either interpretation, study findings underscore the importance of low-barrier and accessible governmental financial supports to prevent the exclusion of structurally marginalized groups, especially during a pandemic. Accordingly, interventions like removing taxable income requirements and the full decriminalization of sex work can help increase access to income supports for people engaged in sex work [9, 31]. Additionally, as highlighted by previous research, offering a universal basic income may be a longer-term approach to reducing the need for engagement in sex work among people engaged in survival sex work [43, 44].

There are several limitations associated with this study. As a result of the cross-sectional nature of this study, it is not possible to make causal inferences. Unmeasured confounding may exist between the explanatory variables and the outcome of interest. For example, the relationship between sex work engagement and receipt of COVID-19 emergency funds may be impacted by pandemic restrictions. Specifically, the decline in sex work engagement during the pandemic may have resulted from public health restrictions, and this decline would have been observed irrespective of access to emergency funds. Additionally, study participants were not a random sample; therefore, findings may not be representative of all PWUD locally or beyond the context in this setting. All data within the study is self-reported, which may lead to recall or social desirability bias; however, previous research has determined that self-reported drug use behaviours are valid and reliable [45]. An additional limitation is that engagement in sex work was broadly defined as exchanging sex for money, goods, drugs, shelter, or anything else, which may raise concerns of construct validity. However, as discussed in prior studies, all definitions of sex work involvement generally have limitations [26, 27]. Lastly, given the COVID-19 pandemic, cohort participants without access to phones and the internet and who typically frequented the frontline study office drop-in spaces and/or received reminders for study follow-up through community services, which were more restrictive or closed due to the pandemic, were less likely to have been included in this current study sample. Our findings, therefore, likely capture a relatively lower-risk group of individuals. However, we took several steps to reduce the possible influence of this selection bias, including loaning participants mobile phones to complete remote study assessments.

In summary, the present study found that engagement in sex work appears to have declined during the early period of the COVID-19 pandemic among PWUD. Importantly, those who recently received income support had a reduced odds of engagement in sex work; however, over a longer period of six months, participants engaged in sex work compared to the rest of the sample, received income support at similar rates. Findings suggest that receipt of COVID-19 pandemic income support may have contributed to reductions in sex work engagement among PWUD, though additional investigation is warranted.

## Data Availability

The data used for this study is not publicly available. For further information on the data and materials used in this study, please contact the corresponding author.

## List of abbreviations

PWUD: People who use drugs
CERB: Canadian Emergency Response Benefit
DTES: Downtown Eastside of Vancouver
ARYS: The At-Risk Youth Study
VIDUS: Vancouver Injection Drug Users Study
ACCESS: AIDS Care Cohort to Evaluate exposure to Survival Services
BIPOC: Black, Indigenous, or other Persons of Colour
PO: Prescription opioid
